# Preparation and Characterization of Polyethersulfone/Activated Carbon Composite Membranes for Water Filtration

**DOI:** 10.3390/membranes13120906

**Published:** 2023-12-12

**Authors:** Gunawan Setia Prihandana, Sayed Sulthan Maulana, Rahmat Santoso Soedirdjo, Venni Tanujaya, Desak Made Adya Pramesti, Tutik Sriani, Mohd Fadzil Jamaludin, Farazila Yusof, Muslim Mahardika

**Affiliations:** 1 Department of Industrial Engineering, Faculty of Advanced Technology and Multidiscipline, Universitas Airlangga, Jl. Dr. Ir. H. Soekarno, Surabaya 60115, Indonesia; sayed.sulthan.maulana-2021@ftmm.unair.ac.id (S.S.M.); rahmat.santoso.soedirdjo-2021@ftmm.unair.ac.id (R.S.S.); venni.tanujaya-2021@ftmm.unair.ac.id (V.T.); desak.made.adya-2021@ftmm.unair.ac.id (D.M.A.P.); 2 Department of Research and Development, PT. Global Meditek Utama—IITOYA, Sardonoharjo, Ngaglik, Sleman, Yogyakarta 55581, Indonesia; tsriani@iitoya.com; 3 Centre of Advanced Manufacturing & Material Processing (AMMP Centre), Department of Mechanical Engineering, Faculty of Engineering, Universiti Malaya, Kuala Lumpur 50603, Malaysia; ibnjamaludin@um.edu.my (M.F.J.); farazila@um.edu.my (F.Y.); 4 Centre for Foundation Studies in Science, Universiti Malaya, Kuala Lumpur 50603, Malaysia; 5 Department of Mechanical and Industrial Engineering, Faculty of Engineering, Universitas Gadjah Mada, Jalan Grafika No. 2, Yogyakarta 55281, Indonesia; muslim_mahardika@ugm.ac.id

**Keywords:** polyethersulfone membrane, activated carbon, composite membrane, good health, clean water

## Abstract

Ultrafiltration membrane technology holds promise for wastewater treatment, but its widespread application is hindered by fouling and flux reduction issues. One effective strategy for enhancing ultrafiltration membranes involves incorporating activated carbon powder. In this study, composite polyethersulfone (PES) ultrafiltration membranes were fabricated to include activated carbon powder concentrations between 0 and 1.5 wt.%, with carbon size fixed at 200 mesh. The ultrafiltration membranes were evaluated in terms of membrane morphology, hydrophilicity, pure water flux, equilibrium water content, porosity, average pore size, protein separation, and *E-coli* bacteria removal. It was found that the addition of activated carbon to PES membranes resulted in improvements in some key properties. By incorporating activated carbon powder, the hydrophilicity of PES membranes was enhanced, lowering the contact angle from 60° to 47.3° for composite membranes (1.0 wt.% of activated carbon) compared to the pristine PES membrane. Water flux tests showed that the 1.0 wt.% composite membrane yielded the highest flux, with an improvement of nearly double the initial value at 2 bar, without compromising bovine serum albumin rejection or bacterial removal capabilities. This study also found that the inclusion of activated carbon had a minor impact on the membrane’s porosity and equilibrium water content. Overall, these insights will be beneficial in determining the optimal concentration of activated carbon powder for PES ultrafiltration membranes.

## 1. Introduction

Water is an essential resource for human life in all parts of the world. However, the availability of clean water is becoming scarce due to the increasing population, environmental pollution, and industrial development. Conventional water treatment methods include adsorption, sedimentation, sand filtration, and disinfection prior to use for daily activities. Adsorption is a cost-effective and efficient filtration technique due to its low energy consumption and lack of derived pollution [1]. However, the inadequate pollutant removal capacity and limited reusability of some adsorbent materials hinder further applications of this method [2]. Sedimentation is a chemical treatment process utilizing chemical reagents and produces secondary pollution in the form of large-scale sludge [3]. Sand filtration effectively treats mildly polluted water, but it is unsuitable for handling more severe contaminations [4,5]. Disinfection eliminates microorganisms using chemical agents [6,7]; however, these chemicals can react with dissolved organic matter to produce disinfection byproducts [8]. Membrane technologies are considered a well-established technology for water treatment applications due to their high separation efficiency, low energy consumption, and operational simplicity [9,10,11,12]. Separation membranes can be classified based on their material composition into polymeric and ceramic membranes [13]. Ceramics membranes are mechanically strong and resistant to organic solvent and microbial attack [14,15]. However, most water filtration systems tend to avoid using ceramics as a separation membrane due to their brittleness, high production costs, and complex fabrication process [16,17]. Polymeric membranes, on the other hand, offer high flexibility, low-cost fabrication, and desirable mechanical, thermal, and chemical properties, making them ideal for water treatment applications [18,19,20,21]. Polymers such as polysulfone [21], polyethersulfone (PES) [22], polyamide [23], polyacrylonitrile [24], polyimide [25], and polyvinylidene fluoride (PVDF) [26] are widely used in filtration membrane fabrication. PES is particularly popular due to its excellent chemical and mechanical stability and biocompatibility [27,28]. However, the low hydrophilicity of PES membranes promotes pollutant deposition on the surface, leading to fouling and flux reduction and limiting the further development of PES membranes [29,30]. Numerous studies have aimed to address these challenges through surface coating modifications [31], plasma treatment [32], grafting [33], and physical blending [34,35,36]. Among the various modification techniques, bulk modification by introducing particles to fabricate membranes has gained more recognition due to its simple operation [37].

There are various types of available fillers, such as zeolites, carbon nanotubes, graphene oxide, and activated carbon, that can be used as additives in mixed matrix membrane preparations. As one of these versatile fillers, activated carbon is extensively used for water treatment processes to separate organic and inorganic pollutants from water, owing to its high surface area porosity and controllable pore structure [38,39]. Moreover, activated carbon has significant potential to reduce membrane fouling when integrated into polymeric materials [40]. Shao et al. [41] investigated the use of combined fouling comprising humic acid and activated carbon powder and found that activated carbon decreased the number of contaminants on the membrane surface, resulting in reduced membrane fouling. The effect of incorporating activated carbon into PES-hydroxyapatite-activated carbon (PES-HA-AC) for pure water flux enhancement and irreversible fouling reduction for humic acid and bovine serum albumin was also examined [42]. The results demonstrated that the fabricated PES-HA-AC composite membranes offered improvements in porosity, average pore size, hydrophilicity, and long-term stability compared to the pristine PES membrane, attributable to the introduction of negatively charged functional groups of HA and alternative water transport pathways provided by activated carbon. Wu et al. [43] developed an activated carbon–octafluoropentanol/polyvinylidene fluoride (AC-OFP/PVDF) composite membrane by blending PVDF and functionalized AC-OFP particles through a wet phase-inversion method. In comparison to the unmodified AC/PVDF membrane, the AC-OFP/PVDF membranes displayed higher permeation flux and improved salt rejection, attributed to the enhanced compatibility between AC-OFP and PVDF.

In this study, activated carbon was employed as a filler for preparing PES composite membranes using wet phase-inversion methods. The resulting composite membranes were characterized using analytical tools such as SEM, water equilibrium, pure water flux, equilibrium water content, porosity, average pore size, protein separation, *E-coli* bacteria removal, and water contact angle. The performance of the composite membranes was subsequently evaluated based on pure water flux and bovine serum albumin rejection.

## 2. Materials and Methods

### 2.1. Materials

**Figure 1:**
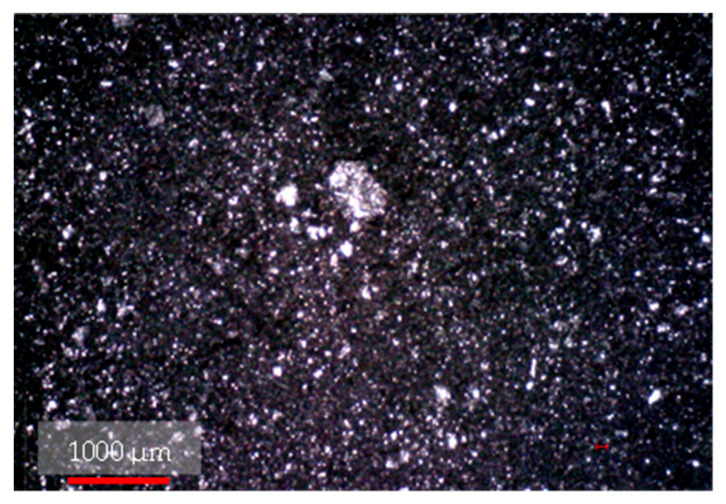
Image of activated carbon powder.

The PES dope solution was prepared using PES 5200, with a molecular weight of 45 (Sumitomo Chemical Co., Ltd., Tokyo, Japan). Bovine serum albumin (66.5) was obtained from HiMedia Laboratories Pvt Ltd., Mumbai, Maharashtra 400086, India. NMethyl-2-pyrrolidone (NMP) was obtained from Merck & Co., Inc., Rahway, NJ, USA. Activated carbon powder of mesh 200 with an estimated size of ~74 µm, as shown in Figure 1, was obtained from a local company in Indonesia. Pure water was used throughout the fabrication process of the membrane and water flux test.

### 2.2. Membrane Fabrication

**Figure 2:**
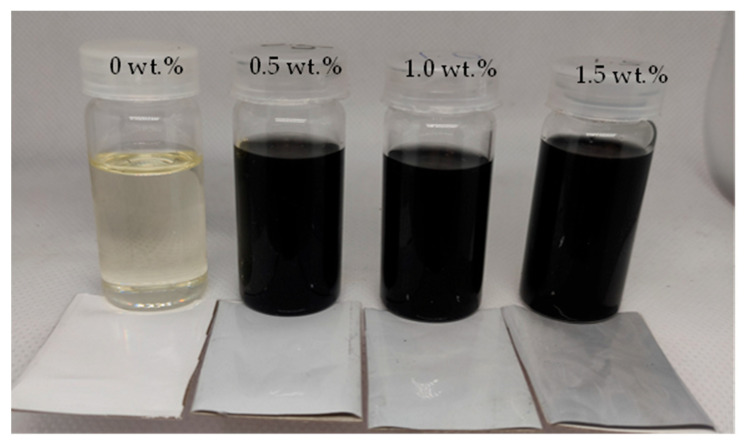
Dope solution and prepared membranes with different activated carbon concentrations.

The PES composite membranes were fabricated using a wet phase-inversion method. PES, as the base polymer, was mixed with the solvent, NMP, until completely dissolved. Once a homogeneous solution was formed, activated carbon powder was gradually added to the dope solution at various concentrations and stirred using a magnetic stirrer. Based on preliminary experiments where the concentration of activated carbon powder at 0.1 wt.% and 0.3 wt.% did not show a significant improvement in water flux, activated powder concentration at an increment of 0.5 wt.% was used in this study, at 0 wt.%, 0.5 wt.%, 1 wt.%, and 1.5 wt.%. The dope solution was then poured onto a glass plate and spread using a film applicator (Elcometer, Manchester M43 6BU, UK) to achieve a wet thickness of 200 microns. Subsequently, the glass plate with the formed solution was carefully transferred to a pure water bath to initiate the coagulation process. Figure 2 presents the dope solution and the fabricated membranes with the respective activated carbon concentrations.

### 2.3. Filtration Experiments

#### 2.3.1. Water Flux Test

**Figure 3:**
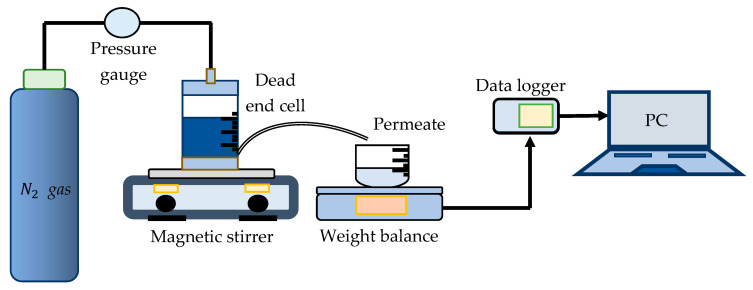
Experimental setup of the water flux test using dead-end cell unit.

The filtration experiment was conducted for 30 min to reach stable flux in a stirred dead-end cell (HP4750 Stirred Cell, Sterlitech Corp. Kent, WA, USA), as illustrated in Figure 3. Nitrogen gas, at a pressure of 2 bar, was introduced into the dead-end cell unit to supply adequate pressure to the pure water. A Weighing Environment Logger recorded the weight of the permeate water traversing the tested membrane. The following equations were used to calculate the volumetric flux $\left(J_{v}\right)$ and permeability $\left(L_{p}\right)$ [44]:

(1) $$J_{v}=\frac{Q}{A\times ∆t}$$

(2) $$L_{p}=\frac{J_{v}}{∆P}$$

where Q is the quantity of the permeate water (in *L*) during the sampling time, ∆t is the sampling time (in h), A is the area of the membrane (in m^2^), and ∆P is the pressure difference (in bar).

#### 2.3.2. Protein Separation

A total of 0.1 wt.% of Bovine serum albumin solution was prepared in phosphate-buffered solution (pH = 7.2). The protein separation experiment was performed at a fixed pressure of 2 bar using a dead-end cell filtration test. An N4S UV-visible spectrophotometer (Ningbo Hinotek Instrument Co., Ltd., Ningbo, China) at a wavelength of 280 nm was used to quantify the concentration of the permeated protein solution. The rejection of solute (SR) was determined by [45,46]:

(3) $$\%SR=\left[1-\frac{C_{p}}{C_{f}}\right]\times 100\;$$

where $C_{p}$ and $C_{f}$ are the permeated and feed solutions of protein concentration, respectively.

#### 2.3.3. Bacteria Filtration Test

Pond water was used as the water source for the bacteria filtration test. Both pond water and irrigation water are considered untreated water sources that may contain contaminants, including the Gram-negative bacteria *Escherichia coli* [47]. Chromocult coliform agar (Chromocult; Merck, Feltham, UK) was prepared following the manufacturer’s guidelines. A total of 26.5 g of Chromocult coliform agar was dissolved in 1 L of pure water. The agar solution was subsequently heated and frequently agitated in boiling water until fully dissolved. After dissolving, the agar was cooled to 45–50 °C before being poured into Petri dish plates to a thickness of 4 mm.

The bacteria colonies of target and test bacteria were prepared by streaking a bacterial suspension onto agar-filled Petri dishes. Water samples were filtered through a fabricated membrane using dead-end filtration cells. The filtered water was then spread across the surface of the agar medium. To ensure even distribution of the bacteria on the agar medium, a T-shaped spreader was used to uniformly streak the bacterial suspension. The culture Petri dishes were incubated at 37 °C for 24 h.

### 2.4. Membrane Characterization

#### 2.4.1. Water Contact Angle Test

A 5 µL drop of pure water was placed onto the surface of the dried membrane using a micropipette. A contact angle image of the dropped water was captured using a digital microscope (Dinolite Edge 3.0 AM73915MZTL, AnMo Electronics, New Taipei City, Taiwan). The angle of the water droplet was calculated using CAD software. To ensure accurate results, the contact angles for each membrane were measured three times, from which the average values were calculated. Subsequently, the work adhesion ${(\omega }_{A}),$ which is the surface energy required to drag water from a membrane surface, can be calculated as follows [48]:

(4) $$\omega_{A}=\gamma_{B}(1+\cos\theta )$$

where $\gamma_{B}$ is the water surface tension (7.2 × 10^−2^ N/m) and θ is the contact angle.

#### 2.4.2. Equilibrium Water Content

The produced membranes were trimmed to the required dimensions, submerged in pure water for a 24 h period, and promptly weighed upon the elimination of surplus water from the membrane’s surface. Subsequently, the membranes were dried until a constant weight was achieved, indicating the absence of any residual water. The membrane’s water content was determined from the following equation [49]:

(5) $$\%\mathrm{WC}=\frac{W_{w}-W_{d}}{W_{w}}\times 100\;$$

where $W_{w}$ and $W_{d}$ are the respective weights of the wet and dry membranes.

#### 2.4.3. Porosity

The membrane porosity was analyzed to determine the effect of blending the activated carbon powder on membrane pore size by a gravimetric method using the following equation:

(6) $$\varepsilon \left(\%\right)=\frac{W_{w}-W_{d}}{\rho H_{2}O\times A\times L}\times 100$$

where A is the effective area of the membrane, L is the thickness of the membrane, and $\rho H_{2}O$ is the density of the water (0.998 $g/{cm}^{3}$).

#### 2.4.4. Average Pore Size

The membrane average pore size ($r_{m})$ of the prepared membranes could be calculated by following the Guerout–Elford–Ferry Equation (7), using the porosity and data of pure water flux [50]:

(7) $$r_{m}=\sqrt{\frac{\left(2.9-1.75\varepsilon \right)8\mu_{H_{2}O}\times L\times Q_{H_{2}O}}{\varepsilon \times A\times ∆P}}$$

where ε is the membrane porosity, $\mu_{H_{2}O}$ is the dynamic viscosity of water at room temperature, L is the membrane thickness, $Q_{H_{2}O}$ is the volume of water passing through the membrane per unit time, A is the active area of the membrane for filtration, and ∆P is the transmembrane pressure.

#### 2.4.5. Molecular Weight Cutoff (MWCO)

The MWCO is described as having a linear correlation with the pore size of the membrane [51]. The evaluation of membrane MWCO involves identifying the smallest inert solute exhibiting a protein rejection of 80–100% in an ultrafiltration test. In this study, a high-molecular-weight protein, bovine serum albumin (BSA), was chosen as the measured protein to indicate the pore size reduction that may occur with the introduction of activated carbon powder.

## 3. Results and Discussion

### 3.1. Pure Water Flux Test Experiments

**Figure 4:**
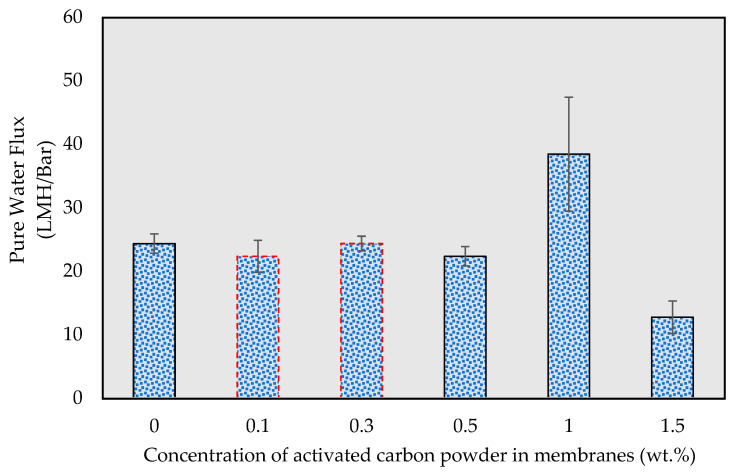
Pure water flux of the membranes at different activated carbon concentrations.

Figure 4 displays the water flux of fabricated membranes with varying concentrations of activated carbon powder. In comparison to the flux of the pristine PES membrane (10.8 LMH/Bar), the water fluxes of the PES composite membranes initially increased and subsequently decreased as the concentration of activated carbon powder grew. The water flux of the PES composite membrane reached its peak value of 38.4 LMH/Bar with an activated carbon powder concentration of 1.0 wt.%, and declined by 33% to 12.7 LMH/Bar as the concentration of activated carbon increased to 1.5 wt.%. This outcome aligns with previous research, which suggested that a rise in water flux is due to an increase in the pore size and porosity of PES composite membranes, as activated carbon powder is porous [43].

**Figure 5:**
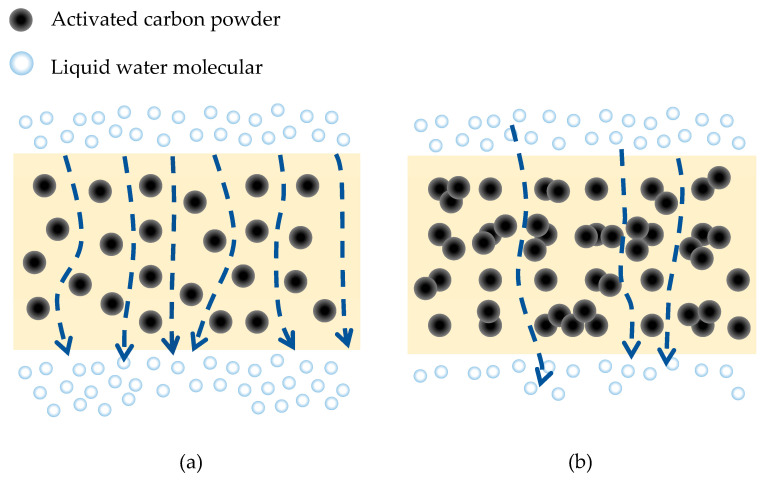
Influence of particle aggregation at different level of concentration on pure water flux. (**a**) low activated carbon concentration, (**b**) high activated carbon concentration.

As depicted in Figure 5a, when the activated carbon powder is distributed uniformly, the carbon powders can facilitate water transfer through their porous multichannel structure. However, as illustrated in Figure 5b, when excessive activated carbon is added, aggregation begins to occur, diminishing the effectiveness of porosity by obstructing the channel entrances and further degrading the mass transfer facilitated by the porous channels of the activated carbon powder. This phenomenon is corroborated by the trend of flux variation observed with the increasing concentration of activated carbon powder in the PES composite membrane.

### 3.2. Protein Separation

**Figure 6:**
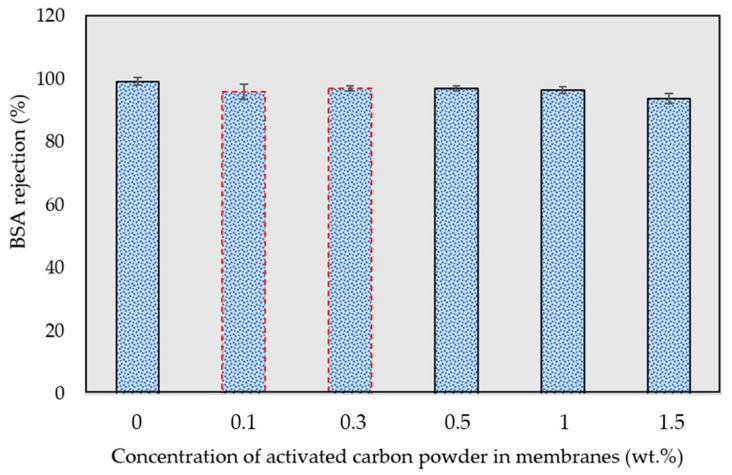
Effect of activated carbon concentration on bovine serum albumin rejection.

Bovine serum albumin (BSA) solution was used to evaluate the fabricated mem-branes’ protein rejection. As presented in Figure 6, due to the high molecular weight of bovine serum albumin, all fabricated membranes rejected over 93% [52]. According to the figure, the PES composite membrane has slightly lower BSA rejection compared to the pristine PSF membrane (0 wt.% of activated carbon powder). However, the BSA rejection of the PES membrane was slightly decreased at a higher concentration of activated carbon powder. In justifying the observed downward trend of an increase in activated carbon concentration, it can be stated that the BSA rejection is not significantly changed at various activated carbon powder weights, since the addition is only up to 2.0 wt.%.

### 3.3. Bacteria Filtration Test

Chromocult coliform agar was chosen for this experimentation to detect E. coli in contaminated surface water [53]. On the chromocult coliform medium, E. coli colonies appear to range from dark blue to violet in color. Background bacteria from the medium are identified as clear or transparent colonies [54]. The E. coli and background bacteria can typically be differentiated by the naked eye.

**Figure 7:**
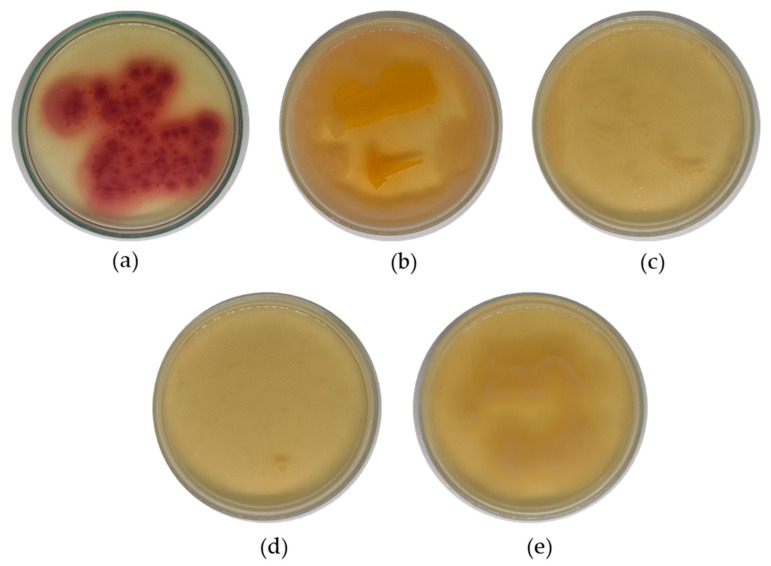
Growth features of *E-coli* on agar medium before and after filtration using membranes with a difference in activated carbon powder concentration: (**a**) polluted water; (**b**) 0 wt.%; (**c**) 0.5 wt.%; (**d**) 1 wt.%; (**e**) 1.5 wt.%.

Figure 7 presents bacterial growth on a chromocult agar medium. The growth of *E-coli* colonies (violet color) from the unfiltered polluted water is clearly visible on the agar plate presented in Figure 7a, indicating that the polluted water contains significant amounts of *E-coli*. Figure 7b–e depict the bacterial growth on agar for filtered water using fabricated membranes containing activated carbon at concentrations of 0 wt.%, 0.5 wt.%, 1.0 wt.%, and 1.5 wt.%, respectively. These figures show the growth of transparent colonies, or background bacteria, from the agar, without any sign of *E-coli* bacteria growth on any of them. This result suggests that both the pristine and composite membranes that were blended with activated carbon powder were successful in filtering *E-coli* bacteria from polluted water.

The fabricated pristine PES membrane in this study is considered an ultrafiltration membrane with a pore size ranging from 5 to 100 nm, efficiently removing bacteria, viruses, and proteins under a low level of applied pressure [55]. Consequently, the incorporation of activated carbon powder into the membrane has no effect on its performance in removing *E-coli* from the polluted water.

### 3.4. Contact Angle Analysis

**Figure 8:**
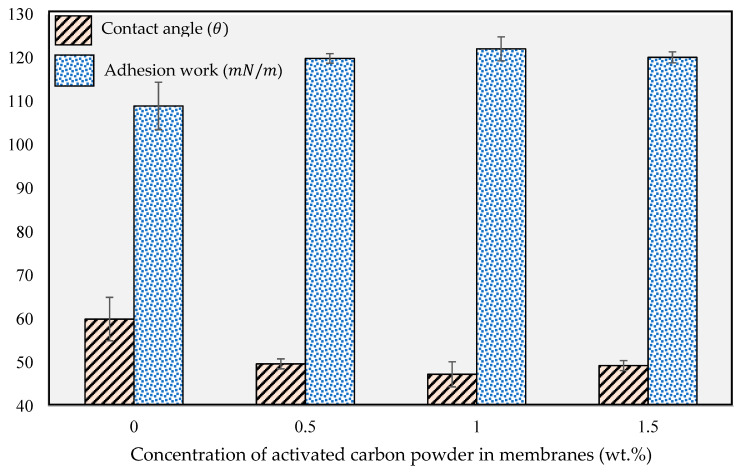
Contact angle and adhesion work of the fabricated membranes at different activated carbon concentrations.

Hydrophilicity is one of the critical factors in evaluating membrane permeability and understanding filtration mechanisms. Water contact angle measurements help in assessing the surface hydrophilicity of fabricated membranes. Generally, hydrophilic membranes display a lower contact angle, with values of less than 90° [56]. Figure 8 shows the measured water contact angles of the fabricated membranes containing 0 wt.%, 0.5 wt.%, 1.0 wt.%, and 1.5 wt.% of activated carbon powder. As depicted in Figure 8, the pristine PES membrane is the most hydrophobic, while the most hydrophilic surface is achieved with a concentration obtained at 1.0 wt.% activated carbon powder.

For membranes with an activated carbon concentration of up to 1.0 wt.%, the water contact angle values slightly decrease with the increase in activated carbon concentration, which is due to the existence of hydroxyl groups in the activated carbon structure [57]. This suggests that, up to a certain concentration, the incorporation of activated carbon powder enhances the membrane’s surface hydrophilicity. However, when the concentration of activated carbon powder in the dope solutions is increased to 1.5 wt.%, significant changes on membrane hydrophilicity occur, most likely due to pore clogging caused by the elevated concentration and agglomeration of activated carbon powder [52,58].

Subsequently, the water contact angle values were used to determine the adhesion work (surface energy) using Equation (4). The acquired values are also presented alongside the water contact angles in Figure 8. The highest contact angle value (60°) and the lowest surface energy (108.9 mN/m) were observed for the pristine PES membrane, while the lowest water contact angle (47.3°) and the highest surface energy (122.1 mN/m) were obtained for the PES composite membrane containing 1.0 wt.% activated carbon powder. This demonstrates that the addition of activated carbon powder at a specific concentration increases the hydrophilicity of the membrane’s surface.

### 3.5. Equilibrium Water Content Study

**Figure 9:**
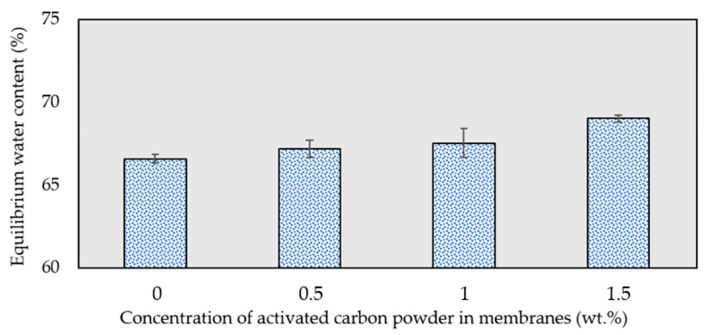
Equilibrium water content of the fabricated membranes at different activated carbon concentrations.

Figure 9 presents the equilibrium water content of the membranes with varying concentrations of activated carbon powder. The figure shows that the water content of the membrane marginally increases as the concentration of activated carbon powder within the membrane rises. Membranes containing activated carbon exhibit a higher water content capacity than the pristine PES membranes, and the capacity for water content in the membranes expands as the concentration of activated carbon powder increases. The interaction between activated carbon and the polymer matrix results in the formation of an interconnected network with porous voids, subsequently increasing the membrane’s water content and further enhancing the membrane’s hydrophilicity [59].

### 3.6. Porosity

**Figure 10:**
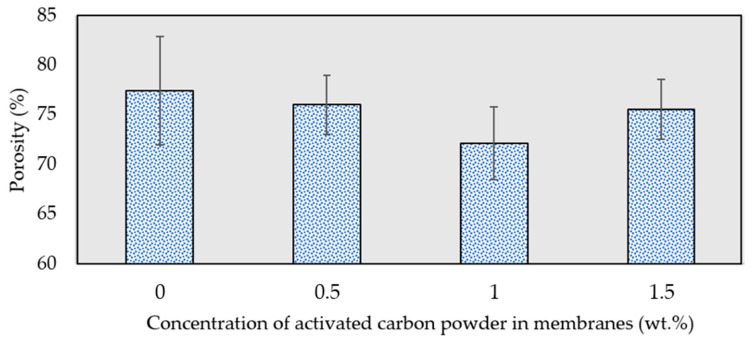
Porosity of the fabricated membranes at different activated carbon concentrations.

Figure 10 illustrates the overall porosity of the membranes. It can be noted that the porosity of the composite membranes decreased slightly in comparison to the pristine PES membrane (0 wt.%). This decline in membrane porosity may be attributed to the increased viscosity of the dope solution, caused by the delayed demixing of solvent and non-solvent during the coagulation process. At higher concentrations of activated carbon powder (1.5 wt.%), the powder presumably functioned as a surfactant, reducing the surface tension of the non-solvent in relation to the polymer film, which consequently led to higher porosity on the membrane surface [60].

### 3.7. Measurement of Average Pore Size

**Figure 11:**
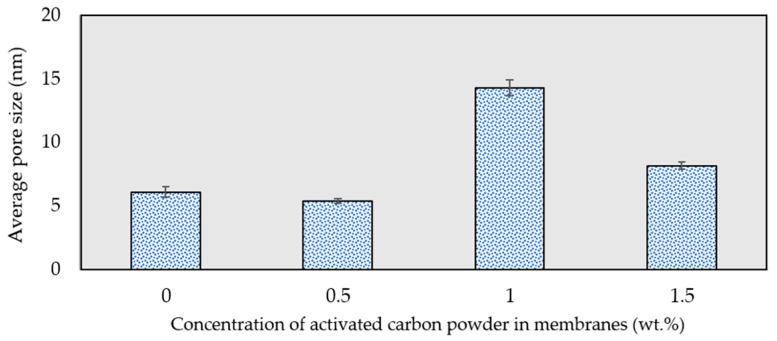
Effect of activated carbon concentration on membranes’ average pore size.

Figure 11 presents the average pore size for all membranes prepared both with and without activated carbon, calculated using Equation (4). The results indicate that the incorporation of activated carbon powder into the membrane matrix prompted an enlargement of the composite membranes’ pore size compared to the pristine PES membrane. An increase in the average pore radius of the composite membranes was observed when the activated carbon concentration ranged from 1.0 to 1.5 wt.%. This behavior aligns with findings reported in the literature [34,61], where the expansion in average pore size can be attributed to increased heterogeneity in the membrane structure, resulting from the integration of activated carbon powder into the dope solution during the phase inversion process. According to the data presented in Figure 11, the calculated average pore size of the membranes decreased at 1.5 wt.% of activated carbon content. This reduction in average pore size can be attributed to the surface pore blockage caused by a higher particle density being dispersed on the membrane surface [62].

### 3.8. Molecular Weight Cutoff Measurement

In this study, bovine serum albumin, with a molecular weight of 69, was used to assess the membrane molecular weight cutoff measurement. The results demonstrated that all membranes exhibited a rejection rate above 90% for bovine serum albumin. This finding indicates that the introduction of activated carbon powder at concentrations of 0.5–1.5 wt.% does not significantly impact the number of pore defects or the overall quality of the membranes [63].

### 3.9. Membrane Morphology

**Figure 12:**
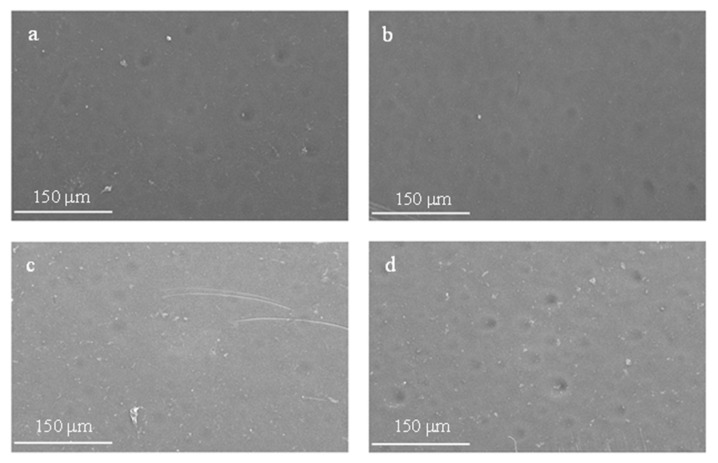
SEM images of the membranes with different activated carbon powder concentrations: (**a**) 0 wt.%; (**b**) 0.5 wt.%; (**c**) 1.0 wt.%; (**d**) 1.5 wt.%.

**Figure 13:**
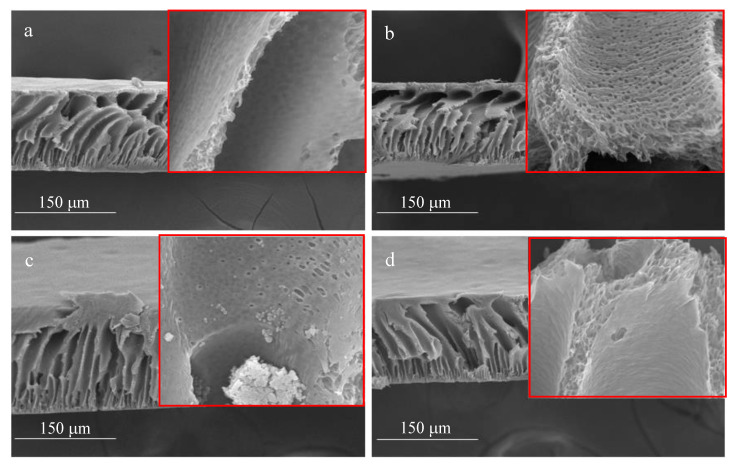
Cross-section SEM images of the membranes with different activated carbon powder concentrations: (**a**) 0 wt.%; (**b**) 0.5 wt.%; (**c**) 1 wt.%; (**d**) 1.5 wt.%.

To obtain a deeper understanding of the fabricated membrane’s performance, the surface morphologies of the membranes were examined using Scanning Electron Microscopy (SEM). The SEM images of the prepared membranes can be seen in Figure 12 and Figure 13. Surface pores with relatively uniform distributions were found for the pristine PES and modified membranes; additionally, a few fine voids were noticed [42]. The water contact angle described in Section 3.1 reveals that membranes with a concentration of 1.0 wt.% are the most hydrophilic amongst all membranes. The scanning electron micrograph in Figure 12c shows that the respective membrane surface is relatively smooth, with fewer craters when compared to the other surfaces, yielding a lower water contact angle. Figure 13 presents a cross-sectional image of the membranes. As depicted in this Figure, the membrane consists of an upper layer where filtration takes place, the middle structure, and the bottom layer. The middle contains a finger-like structure, which supports the upper filtration layer along with the bottom layer. Some of the middle structure walls are filled with microholes, whilst others are relatively smooth and solid. With the incorporation of activated carbon powder, slight alterations in the membrane surface morphology were detected. Although these changes were minimal on the membrane’s top surface, the introduction of activated carbon powder led to considerable alterations in the membrane’s bulk properties and physical appearance, as depicted on their micrographs, yielding a more open structure [64].

## 4. Conclusions

The influence of the addition of activated carbon powder on the performance of PES composite membranes was examined, along with the identification of the optimal concentration for enhancing membrane filtration capabilities. The incorporation of activated carbon powder improved the desirable properties of PES membranes, such as porosity, average pore size, water contact angle, water permeability, protein rejection, and bacterial removal, when compared to pristine PES membranes. An approximate doubling of water flux was observed at an activated carbon concentration of 1.0 wt.%. Additionally, the most hydrophilic surface was observed at this concentration, where the water contact angle decreased by 13°. The bovine serum albumin filtration test indicated that all prepared membranes demonstrated good protein rejection (93–95% at 2 bar). Regarding bacterial (*E-coli*) filtration, the incorporation of activated carbon did not affect the membrane’s performance in removing *E-coli* from polluted water. In conclusion, the experimental results suggest that activated carbon powder holds potential as a significant additive for enhancing the properties of PES membranes in water filtration applications.

## Data Availability

The data presented in this study are available on request from the corresponding author.
